# Identification of malaria parasite-infected red blood cell surface aptamers by inertial microfluidic SELEX (I-SELEX)

**DOI:** 10.1038/srep11347

**Published:** 2015-07-01

**Authors:** Christina M. Birch, Han Wei Hou, Jongyoon Han, Jacquin C. Niles

**Affiliations:** 1 Department of Biological Engineering, Massachusetts Institute of Technology, 77 Massachusetts Avenue, Cambridge, MA 02139, USA; 2 Department of Electrical Engineering and Computer Science, Massachusetts Institute of Technology, 77 Massachusetts Avenue, Cambridge, MA 02139, USA; 3 BioSystems and Micromechanics (BioSyM) IRG, Singapore-MIT Alliance for Research and Technology (SMART) Centre, 1 Create Way, #04-13/14 Enterprise Wing, Singapore 138602, SINGAPORE

## Abstract

*Plasmodium falciparum* malaria parasites invade and remodel human red blood cells (RBCs) by trafficking parasite-synthesized proteins to the RBC surface. While these proteins mediate interactions with host cells that contribute to disease pathogenesis, the infected RBC surface proteome remains poorly characterized. Here we use a novel strategy (I-SELEX) to discover high affinity aptamers that selectively recognize distinct epitopes uniquely present on parasite-infected RBCs. Based on inertial focusing in spiral microfluidic channels, I-SELEX enables stringent partitioning of cells (efficiency ≥ 10^6^) from unbound oligonucleotides at high volume throughput (~2 × 10^6^ cells min^−1^). Using an RBC model displaying a single, non-native antigen and live malaria parasite-infected RBCs as targets, we establish suitability of this strategy for *de novo* aptamer selections. We demonstrate recovery of a diverse set of aptamers that recognize distinct, surface-displayed epitopes on parasite-infected RBCs with nanomolar affinity, including an aptamer against the protein responsible for placental sequestration, *var*2CSA. These findings validate I-SELEX as a broadly applicable aptamer discovery platform that enables identification of new reagents for mapping the parasite-infected RBC surface proteome at higher molecular resolution to potentially contribute to malaria diagnostics, therapeutics and vaccine efforts.

Malaria is an infectious disease for which nearly half of the global population is at risk. There are an estimated 219 million cases and 660,000 deaths annually^1^, due mostly to infection by *Plasmodium falciparum*, which can cause several life-threatening severe complications including placental and cerebral malaria. While the pathophysiology underlying severe malaria is not fully understood, parasite-derived ligands displayed on the surface of infected RBCs have been shown to interact with receptors on host endothelial cells, uninfected RBCs and platelets. These molecular interactions contribute to the sequestration of parasite-infected RBCs within the host microvasculature, which is believed to be a key step in the etiology of severe malaria^2^,^3^. Thus, basic knowledge of the molecular determinants on the surface of parasite-infected RBCs is important for understanding malaria pathogenesis and potentially modulating disease severity.

The variable *P. falciparum* erythrocyte membrane protein-1 (PfEMP1) family, of which there are ~60 *var* gene members, is the most extensively studied protein family on the parasite-infected RBC surface^4^. Several members are clearly linked to severe malaria, including *var*2CSA in placental malaria^5^,^6^ and a subset of group A *var* genes in cerebral malaria^7^,^8^,^9^. While parasite-induced changes in the variety of ligands displayed on the surface of infected RBC surface is recognized as an important mechanism mediating severe malaria, a detailed characterization has been impeded by limitations in the technologies available for interrogating the infected RBC surface proteome. Bioinformatics approaches based on detecting signature ‘PEXEL’ motifs involved in trafficking proteins to the RBC compartment have been used to predict a relatively large number of putatively exported proteins^10^,^11^,^12^. However, not all exported proteins are captured by this analysis, as some PEXEL-negative proteins (PNEPs) are also trafficked to the RBC^13^,^14^. Furthermore, it is not possible to determine *a priori* which of these parasite-exported proteins will be displayed on the infected RBC surface^13^,^14^. Mass spectrometry-based proteomics intended to selectively and directly interrogate the *P. falciparum* infected RBC surface have revealed a limited number of candidates, but surface localization for the majority of these has not been established^15^. However, many non-exported protein contaminants were present within the putatively surface-protein enriched fraction, thus compromising how confidently surface localization can be assigned. Furthermore, known surface-expressed proteins, such as PfEMP1, while detectable by Western blot are under-represented in the dataset. Thus, this approach likely underestimates and in some instances misrepresents the repertoire of parasite proteins present on the infected RBC surface.

Specific affinity reagents, such as antibodies, can be powerful for determining the cell surface localization and, through pull-down coupled with mass spectrometry, the identity of target proteins. Only a limited number of antibody reagents–mostly to a subset of PfEMP1 proteins–are available. However, even when robust antibody reagents are identified, these are available in limited quantities and relatively costly to replenish. We have sought to address this challenge by establishing a new strategy for increasing access to diverse sets of nucleic acid aptamer affinity reagents capable of recognizing distinct epitopes on the surface of parasite-infected RBCs. We reason that aptamers could be ideal affinity reagents to develop as they can: (1) bind their targets with similarly high affinities and specificities as antibodies; (2) be produced inexpensively *in vitro*; and (3) be identified through an *in vitro* selection process (SELEX)^16^,^17^ using live, whole parasite-infected RBCs as targets. Importantly, the latter ensures that recovered aptamers inherently recognize their diverse binding partners when expressed in their native context and natural abundance on the complex parasite-infected RBC surface. An important challenge inherent to this approach, however, is that many distinct targets will be present on the cell surface during selection, and these will drive enrichment of diverse aptamer solutions. Therefore, to prevent high false discovery rates that would compromise efficient recovery of high affinity aptamers, our proposed strategy demands a sufficiently stringent selection process to minimize enrichment of non-binding sequences.

Here, we introduce an aptamer selection strategy referred to as inertial microfluidic SELEX or “I-SELEX” as a novel solution to this challenge. We integrate particle focusing using inertial microfluidics in curvilinear channels^18^,^19^,^20^,^21^,^22^ and SELEX principles to establish a system for continuous partitioning of cell-bound aptamers away from unbound nucleic acids in the bulk solution phase with high efficiency. We first validate this strategy using a model cell target system to characterize the high partitioning efficiency achievable with our device and demonstrate successful *de novo* I-SELEX to identify high affinity aptamers. Finally, we use I-SELEX with unmodified, live parasite-infected RBCs to identify aptamers that preferentially interact with distinct surface protein targets. Overall, I-SELEX represents an efficient strategy for creating a diverse set of aptamer affinity reagents that can be used to more finely map the surface of *P. falciparum* infected RBCs. Given its generality, I-SELEX should also be broadly useful for other SELEX applications using bead-immobilized or live, unmodified whole cell targets.

## Results

### I-SELEX microfluidic device overview

**Figure 1:**
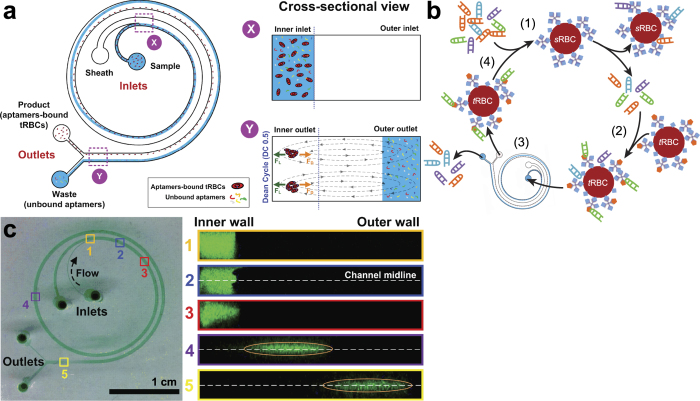
The I-SELEX microfluidic device and its use in aptamer selection is schematically illustrated. (**a**) The microchannel design consists of a bi-loop spiral of radius ~1 cm with dual inlets and outlets. Pre-incubated bead/cell target-aptamer library mixtures and sheath buffer are pumped through the inner and outer inlets of the device, respectively. Under the influence of Dean drag forces (F_D_), unbound aptamers migrate along Dean vortices towards the outer wall and are diverted to the waste outer outlet. Target beads/cells (and any bound aptamers) experience additional strong inertial lift forces (F_L_) and are focused along the inner microchannel wall and collected in the product inner outlet. (**b**) A schematic of the I-SELEX procedure showing: (1) negative selection of random library against *s*RBCs; (2) positive selection of surviving library on *t*RBCs; (3) partitioning of *t*RBC-bound aptamers from unbound library in the I-SELEX device; (4) recovery, RT-PCR amplification and *in vitro* transcription to enrich *t*RBC-bound aptamers. (**c**) Optical cross sections through the device were taken at positions 1–5 along the length of the channel using high-speed confocal microscopy. Fluorescently labeled CRP aptamers injected via the sample inlet enter the microchannel nearest the inner wall (positions 1 and 2). Aptamers injected via the sample inlet are pinched tightly at the inner wall (position 1). Free aptamers begin to migrate across the channel as a tight band along the midline (positions 2-4) and exit the device when they reach the outer wall (position 5).

The device used for I-SELEX is shown schematically (Fig. 1a). The user interface is simple, requiring only a pair of syringe pumps for routine operation. Importantly, the current device is used strictly as a generic strategy for achieving the partitioning step in SELEX (Fig. 1b). It is easily integrated with existing sample processing, and library manipulation and deconvolution procedures. Operationally, the nucleic acid library-target mixture and a sheath buffer are introduced via the inner and outer inlets of the device (Fig. 1a), respectively, at an empirically determined appropriate flow rate. Once steady flow conditions are achieved, cells/particles (and any bound aptamers) are recovered at the product inner outlet, while the non-binding fraction of the nucleic acid library is diverted to the waste outer outlet (Fig. 1a and Supplementary Video S1). The flow profile is stable indefinitely once established, and permits continuous input mixture fractionation and sampling of complex libraries. Under typical operating conditions, a library size of ~10^14^ can be sampled and partitioned in ~10 minutes at a sample flow rate of 150 μL min^−1^ and ~2 × 10^6^ cells min^−1^.

### Fluid mechanics principles governing the design and operation of the I-SELEX device

Due to centrifugal acceleration in curvilinear channels, faster-moving fluid at the channel center moves towards the outer wall in a radial direction along the channel midline. Conservation of mass principles dictate that fluid near the outer channel walls circulates inwardly, which creates two symmetrical and counter-rotating Dean vortices perpendicular to the main axial flow in the channel^23^. The magnitude of these Dean vortices is determined by the dimensionless Dean number parameter (*De*), which relates channel dimensions, curvature, and flow rate as described in Equation (1)^24^:
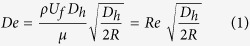
where *ρ* is fluid density (kg m^−3^), *U*_*f*_ is the average primary channel velocity (m s^−1^), *D*_*h*_ is the microchannel hydraulic diameter defined as *2w* × *h/(w* + *h)*, *μ* is fluid viscosity (kg m^−1^ s^−1^), *R* is the radius of curvature, and *Re* is Reynolds number. Ookawara *et al.* later formulated an empirical expression for the average Dean velocity (U_*De*_) for a given *De* as^25^:
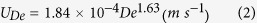


Due to transverse Dean flows, particles flowing in a curvilinear channel experience lateral drag forces (*F*_*D*_), which allow them to migrate across streamlines. Dean drag increases in magnitude with particle size and channel width^26^. We can define the lateral distance traversed by a particle due to Dean flow in terms of ‘Dean Cycle’ (DC). A particle that travels across the entire channel width (*x*-axis) has completed half a Dean Cycle (DC 0.5), and a full Dean cycle (DC 1) upon returning to its starting *x*-coordinate. The path length of a full Dean Cycle (*L*_*DC*_) is approximated by:
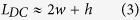
where *w* and *h* are the channel width and height, respectively. Particles may undergo multiple Dean Cycle migrations, the number of which increase directly with channel length (*L*), and flow rate (*Re*). In addition to Dean drag forces, particles in curvilinear microchannels experience an appreciable inertial lift force (*F*_*L*_), which is the combination of a shear gradient lift force (directed toward the channel walls) and a wall-induced lift force (directed away from the channel walls). The superposition of competing inertial lift (*F*_*L*_) and Dean drag (*F*_*D*_) forces results in particle focusing at two equilibrium positions–one within each Dean vortex–provided the particle size (*a*_*p*_) and channel height (*h*) satisfy the following criterion^18^,^27^:
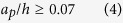


With optimized channel dimensions and flow conditions, these hydrodynamic forces act differentially on particles to achieve highly efficient size-based separation. As the equilibrium position of a particle is strongly dependent on *R*_*f*_, which varies with the third power of particle diameter^28^, this principle has been used to stringently separate mixtures of micron-sized particles^27^,^28^,^29^ or cells^18^. However, inertial focusing of sub-micron sized particles (diameter ≤ 1 μm) and macromolecules is not possible in these devices due to the negligible inertial forces exerted on nanoscale species (*R*_*f*_ < 1). Instead, Dean drag forces dominate, and continuously drive these small species along circulating secondary flows to induce homogeneous mixing. This phenomenon would be undesirable in a SELEX application, as it severely reduces partitioning of the ‘free’ nucleic acid library away from the aptamers bound to the micron-sized bead/cell target. Currently, inertial focusing of molecules is not possible due to difficulties in fabricating sufficiently small microchannels to satisfy the focusing criterion (*a*_*p*_*/h* ≥ 0.07), while tolerating the large pressure drop inherent at high flow conditions in these devices.

We have addressed this limitation by using a two-inlet, spiral channel design (Fig. 1a) in which the sample stream is introduced via the inner inlet while a sheath buffer is pumped via the outer inlet at a higher flow rate to form a tight sample stream at the inner wall. If the channel is truncated at *L* = *n* × (DC 0.5) for *n* ∈ N, circulating macromolecules migrate along the midline (dictated by Dean flow) as a focused band towards the outer channel wall, and are maximally spatially resolved from inertially-focused particles migrating near the inner channel wall (Fig. 1c). Additionally, we built our microchannel with a low aspect ratio (*h/w* << 1) as Dean drag forces become stronger with increasing channel width^26^. The device measures 9 cm (*l*) × 500 μm (*w*) × 60 μm (*h*) with a dual-inlet and dual-outlet spiral microchannel. The product and waste outlet diameters are 150 μm and 350 μm, respectively. We selected the channel height such that particles ≥6 μm in diameter (e.g. human RBCs) predominantly experience inertial forces (*a*_*p*_*/h* ~ 0.1) and focus near the inner channel wall, while macromolecules (e.g. nucleic acid SELEX libraries) experience Dean drag forces (*a*_*p*_/*h* << 0.1) and are transported to the outer channel wall by the time they reach the device outlet (DC 0.5). This allows tightly focused particles/cells (and any bound macromolecules) to be efficiently collected at the product outlet, while unbound macromolecules are diverted to the waste outlet. Primarily, channel height determines particle focusing, and this parameter can easily be varied to accommodate beads/cells of different sizes.

### Whole cell target system used to quantitatively validate the I-SELEX device

We used a synthetic whole cell model to quantitatively characterize the performance of our device during SELEX. We selected human RBCs as a model cell type as they present two extreme yet realistic challenges faced in whole cell-SELEX. First, the cell surface is dominated by a single glycoprotein, glycophorin A, which is present at ~0.5–1 × 10^6^ molecules per cell^30^. This can be used to recapitulate the scenario in complex target whole cell-SELEX where relatively rare surface targets may be occluded by proteins of significantly higher abundance. Second, RBCs have very high cell surface glycan content, and the majority of these terminate in negatively charged sialic acid residues^30^. Thus, RBCs naturally display abundant glycan targets that favor recovery of low affinity (K_*d*_ ~ μM affinity) aptamers^31^. While preserving the above characteristics, we modified the RBC surface to display human α-thrombin as a target protein. This allowed us to take advantage of the previously described ‘Toggle-25’ thrombin aptamer^32^ to characterize the I-SELEX device, and to stringently exclude unfavorable target characteristics as the primary reason for potential failure of a *de novo* SELEX experiment.

**Figure 2:**
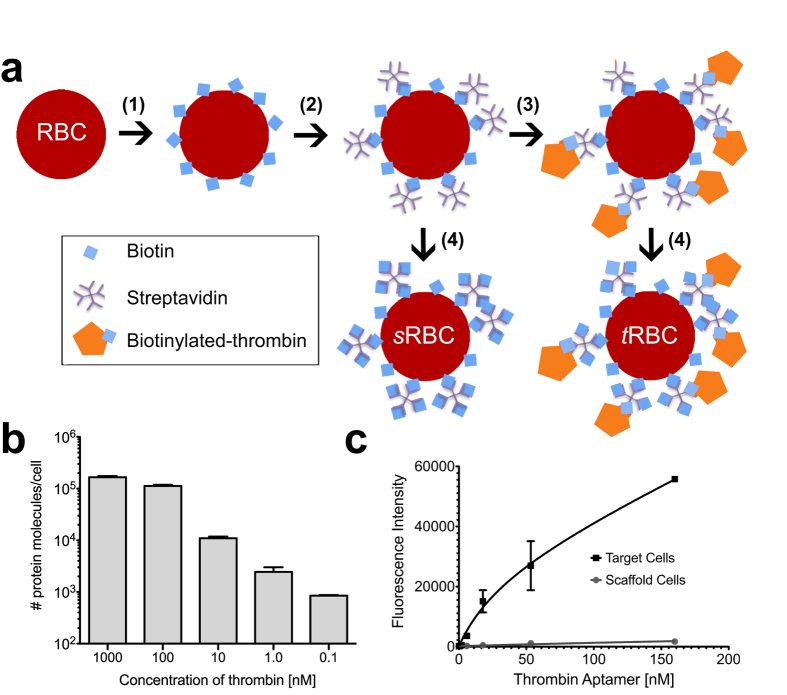
The synthesis and quantitative characterization of a thrombin-displaying model system are summarized. (**a**) Schematic of the process used to create human RBCs that display thrombin from the surface (*t*RBCs) and scaffold-only controls (*s*RBCs). (**b**) Titrated amounts of thrombin can be displayed from *t*RBCs (**c**) Fluorescently-labeled Toggle-25 aptamer selectively binds *t*RBCs but not *s*RBCs.

We first lightly biotinylated RBCs using NHS ester chemistry and coated them with streptavidin to generate ‘scaffold’ RBCs (*s*RBCs) (Fig. 2a). We then bound biotinylated thrombin to the cell surface to produce ‘thrombin-displaying’ RBCs (*t*RBCs). The remaining biotin-binding sites on streptavidin were capped using an excess of free biotin. We determined the final amount of thrombin displayed on *t*RBCs for each new batch prepared, and controlled this parameter by titrating the concentration of biotinylated thrombin used (Fig. 2b). *t*RBCs used in our experiments typically displayed ~10^3^–10^4^ molecules/cell, which is ~50- to 1000- fold lower than glycophorin A. Using 3′-FITC labeled Toggle-25 aptamer, we confirmed that high affinity and specific binding to *t*RBCs occurs (apparent K_*d*_ ~ 34 nM), while no binding to *s*RBCs is observed, as expected (Fig. 2c). This established that the displayed thrombin is accessible and selectively recognized by a cognate nucleic acid aptamer, confirming the suitability of our model system for device characterization.

### The I-SELEX device facilitates resolution of tRBCs and aptamers into distinct streams

We used *tRBCs* and the 5′-FITC labeled C-reactive protein (CRP) DNA aptamer^33^ (no binding to *t*RBCs) to empirically determine the optimal input flow rates needed to simultaneously: *(i)* focus the *t*RBC stream at the inner wall of the device and ultimately into the product outlet; *(ii)* stringently divert the non-interacting CRP aptamer to the outer wall of the device and into the waste outlet; while *(iii)* minimizing *tRBCs* entering the waste outlet. In the limit, these boundary criteria define perfect stringency in the ideal SELEX experiment, where the non-interacting nucleic acid library is completely excluded from the product outlet, and all *t*RBCs with aptamer bound are collected at the product outlet.

**Figure 3:**
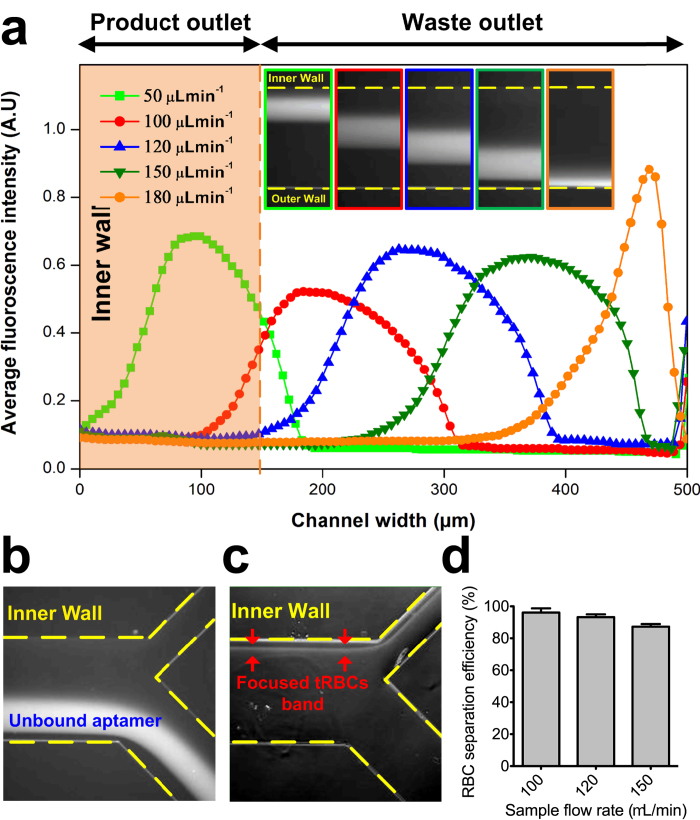
Optimization of the operating flow rates within the I-SELEX device to achieve stringent separation of a model non-interacting aptamer from *t*RBCs while maximizing *t*RBC recovery. (**a**) Average fluorescence intensity line scans showing the normalized distribution of unbound FITC-labeled CRP aptamers (200 nM) across the channel width at increasing flow rates. Approximate positions of the product and waste outlets are indicated. Corresponding fluorescence images illustrating flow positions of unbound aptamers are also shown as an inset (yellow dashed lines indicate the approximate position of the microchannel walls). (**b**) Average composite images indicate unbound aptamers move to the outer wall and are diverted into the waste outer outlet. (**c**) Average composite images indicate efficient *t*RBC focusing to the inner microchannel wall and diversion into the product inner outlet. In both (**b**) and (**c**), the sample input and sheath buffer flow rates are 150 μL min^−1^ and 1500 μL min^−1^, respectively, and the yellow dashed lines indicate approximate positions of the microchannel walls and bifurcation. (**d**) Recovery of *t*RBCs at the product outlet as a percentage of the cells loaded into the device when operated at different sample input flow rates. In all cases, the sheath buffer flow rate is 10-fold higher than the sample input flow rate.

We monitored focusing of the fluorescently labeled aptamer stream by microscopy and empirically determined that predictable control over the free aptamer stream occurs when the sheath buffer flow rate is ten-fold greater than the sample input flow rate. A minimum overall flow rate (sheath buffer + sample) must be maintained (*Re* > 50 or *U*_*f*_ ≈ 900 μL min^−1^) to ensure inertial focusing. At sample input flow rates of 50 μL min^−1^ (total flow rate = 550 μL min^−1^), unbound aptamers are collected in the product outlet with *t*RBCs. As the sample input flow rate is progressively increased to the 180 μL min^−1^ maximum tested (total flow rate = 1980 μL min^−1^), the unbound aptamer is increasingly recovered in the waste outlet (Fig. 3a). Since the diameter of the waste outlet is greater than that of the product outlet, near-complete collection of unbound aptamers in the waste channel can be achieved while inertially focused RBCs remain at the inner wall during their short (<1 s) passage through the device (Fig. 3b and Supplementary Video S1). *t*RBC recovery at the product outlet is inversely dependent on sample input flow rate (Fig. 3c), as increased channel velocity *U*_*f*_ increases the magnitude of Dean drag. Based on these data, we selected a sample input flow rate = 150 μL min^−1^ and sheath buffer flow rate = 1500 μL min^−1^ as standard operating conditions for achieving high partitioning between *t*RBCs and unbound aptamer while maintaining high (~85%) *t*RBC recovery at the product outlet (Fig. 3c).

### The I-SELEX device has a high partitioning efficiency and permits selective enrichment of aptamers from a mock library

**Figure 4:**
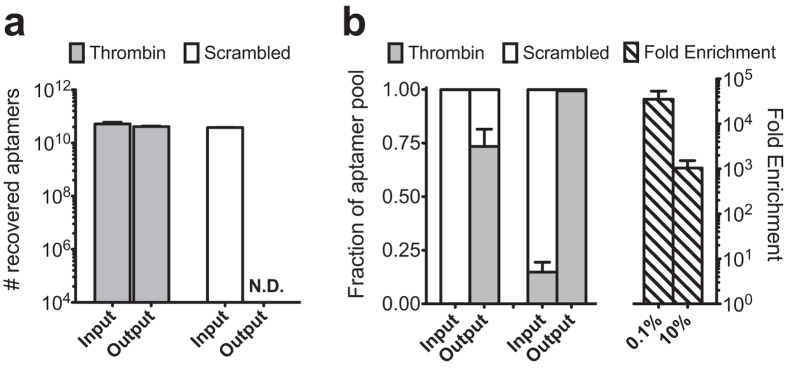
The I-SELEX device exhibits high partitioning efficiency and can be used to selectively recover and enrich target aptamers from mock libraries. (**a**) Comparison of the recovery of Toggle-25 versus *scr*Toggle-25 bound to *t*RBCs targets. (**b**) Enrichment of Toggle-25 aptamers from mock SELEX libraries containing 1:1000 and 1:10 mixtures of Toggle-25: *scr*Toggle-25 using *t*RBC targets during a single pass through the I-SELEX device.

We first tested whether selective recovery of aptamers bound to *t*RBCs can be achieved in our device. We used the Toggle-25 thrombin aptamer and a scrambled version (*scr*Toggle-25) with no affinity for thrombin or *t*RBCs. These were incubated separately at 100 nM with *t*RBCs collectively presenting an ~10-fold excess of thrombin binding sites prior to partitioning using the I-SELEX device. By quantitative RT-PCR, we determined Toggle-25 and *scr*Toggle-25 levels at the sample inlet and product outlet (*t*RBC-bound fraction). Toggle-25 was quantitatively recovered, while the amount of *scr*Toggle-25 collected at the product outlet was below the limit of detection (Fig. 4a).

From these data, we determined the partitioning efficiency (PE) attainable with the I-SELEX device. PE is a common metric used to evaluate SELEX washing methods^34^,^35^, and is a measure of the device’s ability to reject non-binding sequences from the recovered pool containing true aptamers. It is defined as the ratio of the number of input sequences to the number of non-binding sequences recovered in the product output after partitioning. A single pass through our device reproducibly removed ≥10^6^ non-binding sequences (Fig. 4a), establishing this as the lower limit of PE for the I-SELEX device. This PE is similar to or exceeds that attained in NECEEM^34^ and M-SELEX^35^ methods, and is consistent with the reproducibly high PEs accessible using microfluidics.

In preparation for conducting a *de novo* SELEX experiment, we empirically tested whether *t*RBC targets could significantly enrich Toggle-25 thrombin aptamers from a mock library containing excess *scr*Toggle-25. Two artificial SELEX libraries were prepared, each containing ~10^14^ total molecules, and either 0.1% or 10% Toggle-25. Each library was incubated with *t*RBCs (10^7^ cells; 10^4^ thrombin molecules/cell) and this mixture was subjected to a single partitioning step through the I-SELEX device. Significant and preferential enrichment of Toggle-25 was achieved, and the recovered pools from both mock libraries were dominated by Toggle-25 (Fig. 4b).

### Successful *de novo* selection using the I-SELEX device

**Figure 5:**
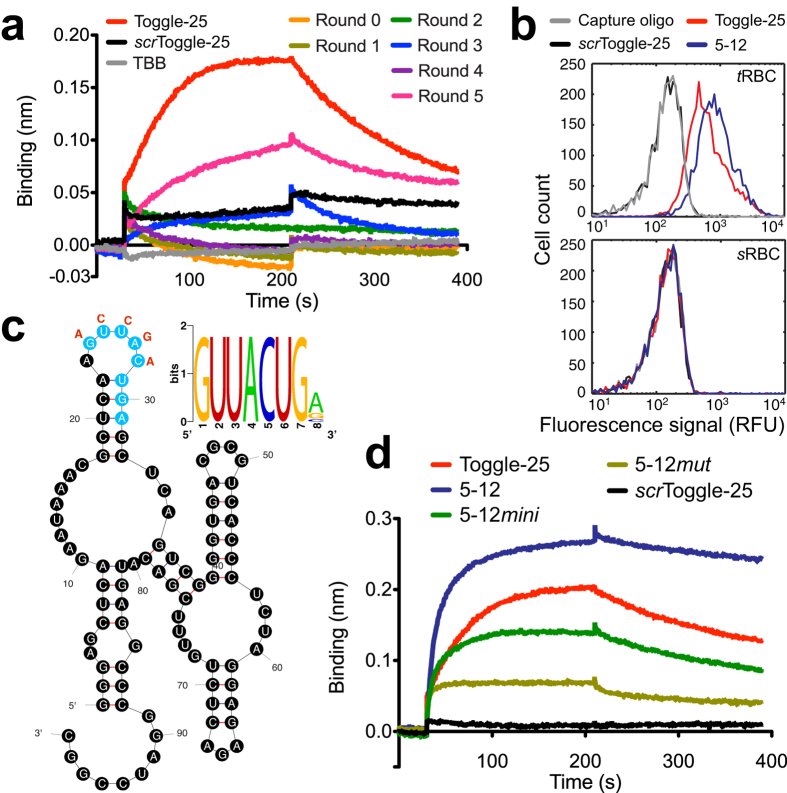
The I-SELEX device can be used for *de novo* discovery of high affinity aptamers. (**a**) Bio-layer interferometry kinetic binding data for the interaction between probe-immobilized thrombin and RNA pools from indicated selections rounds. Toggle-25 and *scr*Toggle-25 were included as positive and negative controls, respectively. (**b**) Comparison of fluorescently labeled 5–12, Toggle-25, *scr*Toggle-25 and capture oligonucleotide binding to *t*RBCs versus *s*RBCs. (**c**) A conserved motif present in Round 3 and 5 selected pools is indicated, and its location within a stem-loop element within the secondary structure predicted for aptamer 5–12 is indicated. (**d**) Evaluation of the importance of this conserved motif to 5–12 thrombin binding affinity was assessed by measuring thrombin binding affinity of: (i) 5–12*mini*, in which the 5–12 was truncated while preserving the motif; and (ii) 5–12*mut*, in which the motif was mutated in the context of full-length aptamer.

Using a randomized library containing ~10^14^ different sequences, we conducted *de novo* SELEX using *t*RBCs as a whole cell target. For each round, the partitioning step was achieved in a single pass of the *t*RBC-library mixture through the I-SELEX device. *t*RBCs were collected at the product outlet, and the bound RNA recovered, amplified by RT-PCR then transcribed *in vitro* using standard procedures in preparation for the next round. A total of five rounds of selection were completed. The bulk initial library and the Rounds 1–5 selected pools were evaluated for thrombin binding by bio-layer interferometry using biotinylated thrombin immobilized on streptavidin probes. The Round 5 selected pool exhibited thrombin binding (K_*d*_ ~ 4 nM), similar to Toggle-25 (Fig. 5a).

We sequenced 26 and 17 clones from the Rounds 3 and 5 selected pools, respectively (Supplementary Table S1), and analyzed these sequences using MEME^36^. The majority (86%) of the sequences were unique. One clone (3–19) was duplicated, and another was represented four times (3–7, 5–3, 5–9 and 5–16). Clones could be broadly grouped into three clusters based on the presence of a MEME-identified motif or lack thereof. Cluster I sequences contained a conserved *GUUACUG* (*A*/*G*/*C*) motif (Motif 1), and 2/25 and 10/13 (31% overall) of the unique clones recovered from rounds 3 and 5, respectively, fell into this group. Clones 5–5 and 5–12, arbitrarily chosen as representatives from this cluster, were identified as high affinity thrombin aptamers by bio-layer interferometry (K_*d*_ ~ 2 nM for both). We tested 5–12 binding to *t*RBCs and *s*RBCs. Similar to Toggle-25, 5–12 exhibited significant binding to *t*RBCs but not sRBCs as expected based on their thrombin-specific binding properties (Fig. 5b). To understand whether Motif 1 is involved in binding, we used secondary structures predicted by mfold^37^ to guide the design of truncated or mutated versions of 5–12 (Fig. 5c). Motif 1 is predicted to lie within a stem-loop region of these aptamers. We generated 5–12*mini* by truncating the parent aptamer while preserving the predicted stem-loop structure containing the conserved motif, and determined that high-affinity thrombin binding was retained (K_*d*_ ~ 5 nM). In the context of the full-length aptamer, we mutated the motif while preserving the stem-loop element to produce 5–12*mut*. This mutated aptamer exhibited significantly reduced thrombin binding (K_*d*_ ≥ 3 μM) (Fig. 5d). Taken together, these data indicate that the conserved motif present in Cluster I aptamers may be required for high affinity binding to thrombin.

A sequence identical to Toggle-25 was not present amongst the clones sampled, and Motif I is distinct from that implicated in Toggle-25 binding to thrombin^32^. However, Cluster II clones contained a *UU[G/C/A]YCC[A/U]AG* motif (Motif 2) that is shared between Toggle-25, 3–4, 3–10, 3–19 and 5–6. The representative 3–19 exhibited very low binding affinity to thrombin (≥1 μM). Thus, while this conserved feature may confer some degree of binding to thrombin, it appears insufficient for mediating the high affinity binding observed for Toggle-25. Cluster III consists of sequences that do not contain the above motifs or another sequence feature that is strongly conserved amongst the group. We tested 3–7, 3–9 and 3–12 for thrombin binding. Although 3–7 was represented by four separate clones, it did not detectably bind thrombin. It is possible that this sequence persisted due to a replication advantage rather than target-binding affinity, or that Clone 3–7 requires the full tRBC context for high-affinity binding. Clone 3–12 bound thrombin with modest affinity (K_*d*_ ~ 200 nM ), while 3–9 bound with high affinity (K_*d*_ ~ 4 nM). Altogether, using I-SELEX with a complex whole cell target, we have identified a new class of high-affinity binding thrombin aptamers that are sufficiently enriched from a starting library of ~10^14^ sequences within three rounds of selection. It is also worth noting that, while the I-SELEX partitioning step is stringent, aptamers with affinities varying by 100-fold (~2–200 nM) can be recovered. Thus, without having to devise alternative partitioning protocols, I-SELEX may be broadly useful in recovering both high and modest affinity aptamers to a given target, thus expanding the option space for identifying the most suitable aptamer for the intended application.

### Selecting aptamers against the surface of malaria parasite-infected red blood cells using I-SELEX

We used the same library (with ~10^14^ diversity) as above in a *de novo* I-SELEX experiment in which RBCs infected with the *P. falciparum* CS2 strain were used as whole cell targets. The CS2 strain stably expresses a single PfEMP1 variant, *var*2CSA, which is associated with placental malaria, and does not undergo the normal process of switching which *var* gene is expressed^5^,^38^,^39^,^40^. We designed our selections to recover aptamers capable of preferentially interacting with any molecular feature(s) present on CS2-infected but not uninfected RBCs. This was achieved by negatively selecting the library on uninfected RBCs and positively on parasite-infected RBCs during each round. In SELEX Rounds 6–8, we pursued two selection schemes in parallel. In scheme 1, we increased selection stringency through a 5-fold dilution of the positive selection mixture relative to earlier rounds, while in scheme 2, we maintained the same level of stringency as in Rounds 1–5.

**Figure 6:**
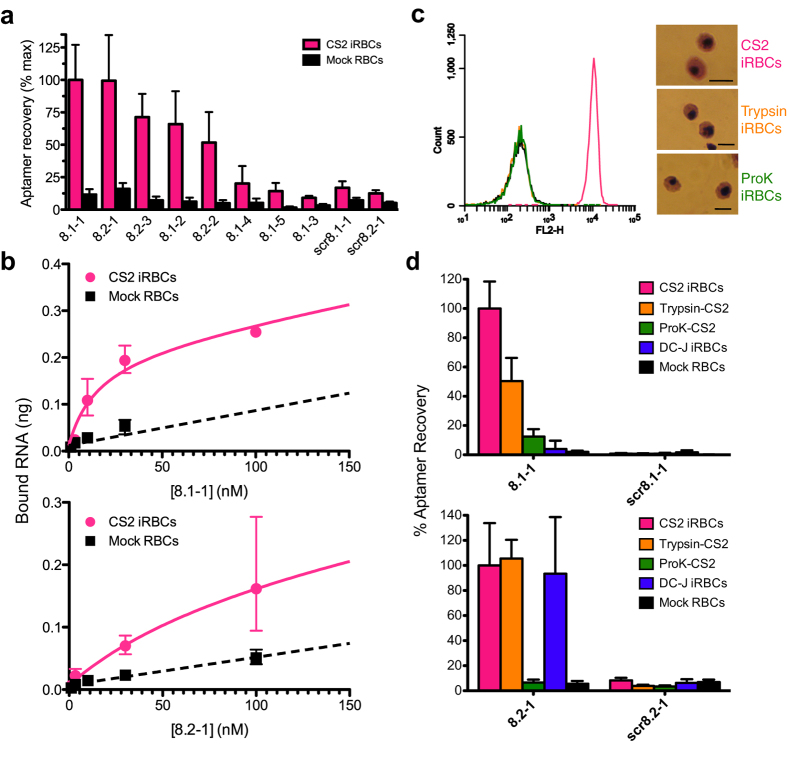
Characterization of aptamers against malaria-infected red blood cells generated via I-SELEX. (**a**) Relative binding of aptamers isolated from Round 8 of selection 1 (8.1-x) or selection 2 (8.2-x) to CS2 infected RBCs versus uninfected, mock-cultured RBCs. This is plotted as the amount of aptamer recovered for the particular cell type at the sample outlet of the I-SELEX device. (**b**) Binding isotherms for aptamers 8.1-1 and 8.2-1 to CS2 infected RBCs (magenta line), with non-specific binding to mock cultured, uninfected RBCs indicated (black dotted line). (**c**) Glycophorin A (GPA) levels present on the surface of untreated or protease treated RBCs, and Giemsa-stained light microscopy images of the corresponding cells. Scale bar: 8 μm. (**d**) Profiling of aptamers 8.1-1 and 8.2-1 against CS2 infected RBCs (protease treated or untreated) and the DC-J parasite lines that do not express PfEMP1 surface proteins.

After eight rounds of selection, we determined a limited number of sequences from the enriched pools (16 and 17 from schemes 1 and 2, respectively). Approximately two-thirds of the sampled sequences were unique, and no strongly conserved motifs amongst these could be detected by MEME^36^. However, several sequences were represented multiple times (Supplementary Table S2). From scheme 1, 8.1-1 and 8.1-2 were represented four and two times, respectively, while in scheme 2, 8.2-1 and 8.2-2 were each represented three times. We selected eight sequences total (8.1-1,2,3,4,5 and 8.2-1,2,3) and determined that these all selectively bound CS2 parasite-infected RBCs over mock cultured uninfected RBCs (Fig. 6a; Supplementary Fig. 1). Sequences clustered into high recovery (8.1-1,2 and 8.2-1,2,3) and low recovery (8.1-3,4,5) groups, with all multiply represented sequences (8.1-1, 8.1-2, 8.2-1 and 8.2-2) clustering in the high recovery group. To test the sequence specificity of aptamer enrichment, we randomized the variable region of the most highly represented sequence from each selection (*scr*8.1-1 and *scr*8.2-1). Both *scr*8.1-1 and *scr*8.2-1 were poorly recovered, similar to 8.1-3 and 8.1-4, and we used these to establish enrichment values (ΔΔCp ~ 1.2-1.3) that define the non-specific binding threshold. We selected two aptamers from the high recovery group, one from each selection scheme, and calculated apparent K_*d*_ values for binding to CS2 parasite-infected RBCs by measuring the amount of recovered aptamer at different concentrations (Fig. 6b). Using the amount of aptamer recovered from mock cultured uninfected RBCs as a measure of nonspecific binding, we calculated apparent K_*d*_ values of ~14 nM and ~84 nM for aptamers 8.1-1 and 8.2-1, respectively.

### Profiling the interaction of aptames with the parasite-infected RBC surface

To understand the nature of the target with which the recovered aptamers interact on the parasite-infected RBC surface, we assessed how recovery of the most represented aptamers from each selection, 8.1-1 and 8.2-1, is affected by limited trypsin or Proteinase K proteolysis of the infected RBC surface. We verified successful proteolysis by monitoring loss of glycophorin A from the cell surface, and confirmed that no gross morphological changes detectable by light microscopy after Giemsa staining had occurred in treated cells (Fig. 6c). Enrichment of 8.1-1, scr8.1-1, 8.2-1 and scr8.2-1 on protease-treated or untreated parasite-infected RBCs target cells was then compared (Fig. 6d). As expected, basal levels of the non-binding controls scr8.1-1 and scr8.2-1 were recovered under the various tested conditions. However, 8.1-1 and 8.2-1 exhibited distinct enrichment profiles on the differently treated target cells. Specifically, 8.1-1 binding showed partial trypsin sensitivity (aptamer recovery reduced by ~50%) and high Proteinase K sensitivity (aptamer recovery reduced by ~90%). Conversely, 8.2-1 binding is trypsin resistant but Proteinase K sensitive (aptamer recovery reduced by ~95%). These data show that our selection process successfully recovered aptamers that recognize either distinct epitopes on the same protein or distinct proteins present on parasite-infected RBCs.

Since our I-SELEX protocol was designed to recover aptamers to any target on the parasite-infected RBC surface, we sought to determine whether any of the identified aptamers could interact with the PfEMP1 protein family. To test this, we used the previously reported DC-J line that has been engineered such that expression of all *var* genes is prevented when parasites are grown under blasticidin-S selection pressure^41^. We then compared enrichment levels of 8.1-1 and 8.2-1 on DC-J (no PfEMP1 expression) and CS2 (*var2*CSA expressing) parasite-infected RBCs. Interestingly, while 8.1-1 is enriched on CS2-infected RBCs, we observed no significant enrichment on DC-J-infected RBCs above background (Fig. 6d). In the case of 8.2-1, however, enrichment on both CS2- and DC-J–infected RBCs were similarly high. Altogether, these data show that 8.1-1 recognizes *var2*CSA on CS2-infected RBCs and its binding to the parasite-infected RBC surface is moderately trypsin sensitive. On the other hand, 8.2-1 interacts with a non-PfEMP1 protein target via an epitope that is resistant to trypsin. Thus, our selection scheme using the I-SELEX strategy facilitates recovery of aptamers capable of recognizing distinct surface epitopes on parasite-infected RBCs.

## Discussion

Here, we have validated a novel and generalizable I-SELEX strategy that is broadly useful for identifying aptamers, and demonstrated its application to model targets displayed on whole cells and live malaria parasite-infected RBCs. We show that this approach facilitates recovery of a diverse set of high affinity and specific aptamers to either a single target artificially displayed on a cell surface or a complex combination of targets natively displayed on whole cells. In applying I-SELEX to malaria parasite-infected RBCs, we show for the first time that this strategy enables discovering novel affinity reagents that recognize distinct surface proteins on infected RBCs, including against the protein responsible for placental sequestration, *var*2CSA. This is achieved without a requirement for first predicting or otherwise defining relevant surface determinants, while circumventing the challenge of obtaining potentially difficult-to-express and natively folded recombinant proteins for aptamer selection. Thus, I-SELEX addresses several key challenges that have limited access to sufficiently diverse affinity reagents to facilitate defining the *P. falciparum*-infected RBC surface proteome in greater detail.

In our *de novo* selections aimed at establishing proof-of-concept for the I-SELEX strategy, we restricted both sequence and binding analyses to a small fraction of the enriched library. We reasoned that the high partitioning efficiency of the device should favor recovery of pools significantly enriched for binders, thus limiting the requirement to deeply sequence and functionally evaluate our selected pools during our validation phase. Our successful proof-of-concept, especially with the malaria parasite-infected RBCs now strongly indicates that systematically analyzing our archived pools from each round of selection by deep sequencing will likely significantly diversify the pool of aptamers capable of specifically recognizing distinct surface epitopes/antigens on infected RBCs. We anticipate that subsets of these aptamers will be important for different basic applications, including use as: capture reagents for pull-down and identification of surface antigens; modulators of parasite-infected RBC interactions with host endothelial and other cell types linked to severe disease pathogenesis; and biomarkers of malaria infection. The information gained through studies in these respective areas is expected to enable translational efforts relevant to formulating blood stage vaccines that include appropriate surface antigens, devising mechanism-based adjuvant therapies to prevent severe malaria disease and developing new malaria diagnostics.

From a technology development standpoint, we have demonstrated application of inertial microfluidics principles in a spiral device capable of rapidly and stringently resolving micron-sized particles (RBCs) from macromolecules (oligonucleotides) to successfully perform whole cell-SELEX. Our device functions with very high partitioning efficiency (≥10^6^) and affords high affinity aptamers in as few as three rounds of selection. We believe this device provides a generic strategy for effectively completing the critical partitioning step in SELEX, and has the advantage of being equally applicable to bead-immobilized targets and unmodified whole cells. The theoretical principles guiding device design and operation are sufficiently well understood such that new designs to accommodate particles of different sizes can be easily achieved. Alternatively, standardization of the partitioning step using a single device design is feasible as the device has sufficient tolerance to accommodate a range of particle sizes with no adverse impact on the ability to resolve particles and macromolecules into distinct output streams.

The microfluidics partitioning strategy we describe takes advantage of the combined effects of inertial focusing of *t*RBCs and well-controlled Dean migration of unbound nucleic acid library along the channel midline in spiral microchannels. By choosing a low-aspect ratio channel design, we significantly enhance the resolving power of our device such that highly efficient separation of micron-sized particles/cells from macromolecules is attainable. Remarkably, such stringent partitioning occurs extremely rapidly, as a particle or cell spends less than a second within the device under our standard operating conditions. We hypothesize that additional particle/cell rotation due to high-shear gradients and secondary Dean flows near the channel wall might play a role in enhancing removal of weakly-bound aptamers from target cells. While cells remained focused near the inner wall, Dean vortices establish a lateral shear gradient at their focusing positions, which may cause cells to rotate as they flow along the channel. This hypothesis is supported by a study showing that the combination of cell rotation and transverse motion in a spiral channel enhanced transfection efficiency via more homogenous electroporation of individual cells^42^. By varying these parameters, we can potentially tune the degree of Dean flow-induced target cell/particle surface “washing” achieved during I-SELEX. This could potentially allow selection stringency to be flexibly modulated.

In summary, we have designed, validated and used a simple and broadly applicable inertial microfluidics device as an efficient way of achieving stringent, single-pass library partitioning during I-SELEX. Using this strategy, we have identified a diverse set of aptamers, and shown that a subset of these specifically interact with distinct epitopes uniquely present on malaria-parasite infected red blood cell surfaces. We envision these aptamer reagents will be useful for characterizing the malaria parasite surface proteome in greater molecular detail, which would in the long-term potentially benefit efforts to develop malaria diagnostics, adjunctive treatment, and vaccines to both reduce mortality and prevent the disease.

## Methods

### Device fabrication and flow conditions

Microfluidic devices were fabricated in polydimethylsiloxane polymer (PDMS, Sylgard 184, Dow Corning, USA) using the double molding process reported previously^43^. Briefly, the patterned silicon wafers were silanized with trichloro (1H, 1H, 2H, 2H-perfluorooctyl) silane (Sigma Aldrich, USA) for 1 hr and PDMS prepolymer mixed in 10:1 (w/w) ratio with curing agent was poured onto the silanized wafer and baked at 80 °C for 1 hr. The cured PDMS mold then acted as a template for subsequent PDMS casting (negative replica). The PDMS master template was silanized for 1 hr before use to aid release of subsequent PDMS microchannels. Finally, holes (1.5 mm) for inlets and outlets were punched and the PDMS microchannels were irreversibly bonded to microscopic glass slides using an air plasma machine (Harrick Plasma Cleaner, USA) and left for 2 hr at 70 °C to complete the bonding.

Fluid flow through the microfluidic device was modulated with two NE-300 Just Infusion™ Syringe Pumps, one for sample input (inner inlet) and one for sheath buffer input (outer inlet) (syringepump.com). Micro-tubing (0.86 mm ID (inner diameter) by 1.52 mm OD (outer diameter)) (Scientific Commodities, Inc.) was used to move fluid from the syringes into the device inlets. Using slightly oversized tubing (OD 1.52 mm > device input/exit punch diameter of 1.5 mm) creates enough friction to hold the tubing in place during routine use at the relevant flow rates. Each sample mixture was pumped into the inner inlet at 150 μL min^−1^ while sheath buffer (Thrombin Binding Buffer or TBB = 20 mM HEPES pH 7.4, 150 mM NaCl, 2 mM CaCl_2_) was pumped through the outer inlet at 1500 μL min^−1^. Sample at the product outlet (cells and aptamers) was collected after 1.5 minutes of run time to allow for establishment of Dean vortices along the channel length.

A new device was used for each I-SELEX experiment. However, the inertial devices can be reused up to 20 times if properly washed. Prior to reuse, devices were washed sequentially with 10 mL TBB, 10 mL water and 2 mL isopropyl alcohol, after which air was pumped into the device to remove all remaining liquid. The device was set on a hot plate (85 °C) for 6–12 hours to solidify the PDMS-glass bond. Before each subsequent use, the device was again washed with 10 mL TBB, a fraction of which (100 μL) was periodically captured and analyzed by qPCR to ensure no sequences were carried over from a prior partitioning.

### Microscopy

Samples (10^7^ cells/mL) and sheath buffer (TBB) were pumped through the microfluidic device using two syringe pumps (NE-1000, New Era Pump Systems Inc., USA) and the ratio between the sample and sheath buffer flow rates was fixed at 1:10. For image acquisition, the microchannels were mounted on an inverted phase contrast microscope (Olympus IX71) equipped with a Hamamatsu Model C4742-80-12AG CCD camera (Hamamatsu Photonics). IPLab (Scanalytics) software was used for video acquisition and captured videos were analyzed using ImageJ^®^ software. A high speed CCD camera (Phantom v9, Vision Research Inc.) was used to capture additional rotational motions of individual focused RBCs within the channels using 1 μs exposure time (~70,000 fps).

### Cell synthesis and quality control

Human RBCs were washed twice with 10 mL PBS, pH 8, and 2 × 10^9^ were resuspended to 5% hematocrit in 4 mL PBS, pH 8 with 1 mg EZLink Sulfo-NHS-Biotin (ThermoScientific). Cells were incubated for 30 minutes rotating at room temperature (25 °C) then washed twice with 10 mL PBS, pH 7.4. Biotinylated RBCs were resuspended to 5% hematocrit in PBS, pH 7.4 and incubated with 200 μg streptavidin (ThermoScientific) for 30 minutes at room temperature. Half of this cell suspension was directly transferred to a fresh tube and incubated with 100 μL of biotin-saturated PBS, pH 7.4 for 15 minutes at room temperature to cap free biotin-binding sites and create *s*RBCs. The other half of the cell suspension was incubated with thrombin-BFPRck (Haematologic Technologies Inc.) for 30 minutes prior to biotin capping to create *t*RBCs. *t*RBCs were synthesized with surface thrombin concentrations spanning 0.1–1000 nM. All cells were stored at 4 °C between use in RPMI-1640 media supplemented with 5 g/L Albumax II (Life Technologies), 25 mM HEPES pH 7.4 (pH adjusted with potassium hydroxide), 2 g/L sodium bicarbonate, 1 mM hypoxanthine, and 50 mg/L gentamicin. Effective surface thrombin concentrations were determined by nonlinear regression analysis of titrated monoclonal anti-thrombin antibody (Haematologic Technologies) labeled with a 1:1000 dilution of Alexa Fluor 488 goat anti-mouse secondary antibody (Life Technologies) before FACS analysis (FL1 channel). Binding data were fit to the following model: *Y* = *B*_max_*X/(X + *K*_*d*_) + NS*X + *Y*_*0*_, where *Y* is fluorescence signal (RFU), *B*_*max*_ is the total number of antibody-accessible binding sites, *X* is the number of antibody molecules added to the solution, NS is the non-specific binding component, and *Y*_*0*_ is the background fluorescence.

### Thrombin aptamer design and synthesis

The DNA template for the thrombin-binding Toggle-25 aptamer^32^ was assembled by PCR using primers CMB49, CMB50, CMB51 and CMB56 (Table S2). *scr*Toggle-25, a non-binding variant was prepared by scrambling the thrombin-binding region of Toggle-25 and installing unique primer binding sites while retaining overall length and (G + C) content. The *scr*Toggle-25 DNA template was PCR-assembled using primers CMB95, CMB96, CMB97 and CMB98. Four aptamer clones from Round 3 were PCR-assembled using the SELEX Forward Primer and the following sequence-specific primers: 3–7 (identical to 5-3), CMB112, CMB113, CMB114; 3–9, CMB107, CMB108, CMB109; 3–12, CMB133, CMB119, CMB120; and 3–19, CMB134, CMB122, CMB123. Three aptamer clones from Round 5 were PCR assembled using the SELEX Forward Primer and the following sequence-specific primers: 5-3 (see above); 5-5, CMB142, CMB125, CMB141; 5–12, CMB126, CMB116, CMB117. Mutant 5–12 (5–12*mut*) templates were synthesized with SELEX Forward Primer and sequence-specific primers CMB130, CMB131 and CMB132. The minimized aptamer (5–12*mini*) was synthesized from the DNA template assembled from the SELEX Forward and CMB127 primers. All PCR reactions were performed identically with Phusion High-Fidelity PCR polymerase (New England BioLabs) according to manufacturer’s instructions, with the exception that internal primers were used at 10-fold lower concentrations than the external primers.

### Partitioning efficiency and enrichment experiments

For partition efficiency experiments, 100 nM of either Toggle-25 or *scr*Toggle-25 was incubated in 1 mL TBB with 10^7^ *t*RBCs for 20 minutes at room temperature with continuous gentle inversion before being passed through the I-SELEX device. *t*RBCs were recovered directly from the sample outlet onto a vacuum filter plate membrane (Millipore MSHVS4510). Bound aptamers were eluted (off-vacuum) by a five minute incubation with 1 mM EDTA in PBS, pH 7.4 followed by plate centrifugation. Quantitative polymerase chain reaction (qPCR) with SYBR Green was used to determine absolute levels of Toggle-25 and *scr*Toggle-25 recovered after device partitioning. Recovered aptamers were ethanol precipitated and reverse transcribed using SuperScript III Reverse Transcriptase (Life Technologies) with either Toggle-25 reverse primer CMB94 or *scr*Toggle-25 reverse primer CMB104. The partner forward primers (CMB77 and CMB106, respectively) were added with 1 × SYBR Green for qPCR quantitation using a Light Cycler 480 (Roche Applied Science). qPCR primers were used at a final concentration of 0.5 μM. The thermocycling program used was as follows: initial denaturation 95 °C for 5 min, followed by 40 cycles of 95 °C for 30 s, 58 °C for 30 s, and 72 °C for 30 s, followed by melting curve analysis. For enrichment experiments, 100 nM total aptamer library (either 90% or 99% *scr*Toggle-25) was incubated with 10^7^ *t*RBCs for 20 minutes at room temperature with continuous gentle inversion before being passed through the device. In these enrichment experiments, where the recovered aptamer sample contained two species, samples were divided in half after recovery and precipitation, followed by aptamer-specific reverse transcription and qPCR, as described above.

### *t*RBC I-SELEX

The DNA template for the random library containing a 50 nucleotide randomized region was chemically synthesized (Integrated DNA Technologies). The primers used for RT and PCR amplification of the library are summarized (Supplementary Table S3). A 2′-fluoro-pyrimidine RNA library containing approximately 3 × 10^14^ unique sequences was synthesized using the DuraScribe T7 Transcription Kit (Epicentre Biotechnologies). RNA was denatured at 75 °C for 5 minutes, followed by refolding for 10 minutes at room temperature. The aptamer library was then incubated with 10^8^ *s*RBCs in 1 mL TBB for 30 minutes at room temperature with continuous gentle mixing. *s*RBCs and the aptamers bound to them were removed by centrifugation. The supernatant was incubated with 10^6^ *t*RBCs for 60 minutes at room temperature with continuous gentle mixing. After incubation, the entire binding reaction was partitioned in a single pass through the I-SELEX device at a flow rate of 150 μL min^−1^ with sheath buffer (TBB) pumped at 1500 μL min^−1^. Cells were recovered and aptamers eluted as described above for enrichment experiments, above. The recovered aptamers were RT-PCR amplified using an empirically determined minimum number of PCR cycles (using the thermocycling protocol listed above), which were minimized to prevent formation of truncation or PCR giant products (14 cycles on average). Half of the recovered PCR product (~1 μg) was stored, and the remainder used for *in vitro* transcription (37 °C for 6–15 hours) to prepare RNA library for the subsequent round. Remaining template DNA was removed by Turbo DNase (Life Technologies). RNA was purified by phenol-chloroform extraction and ethanol precipitation, then re-dissolved in ddH_2_O. Five rounds of selection were performed. DNA from the Round 5 library was cloned into pGEM®-T vectors (Promega), transformed into DH5α cells and mini-prepped plasmid DNA sequenced to identify isolated aptamers (Supplementary Table S1).

### Flow cytometry

To test binding of Toggle-25 to *t*RBCs versus *s*RBCs, Toggle-25 was fluorescently labeled by addition of a single 3′-amino-2′,3′-ddATP (TriLink Biotechnologies) by Poly(A) Polymerase (New England BioLabs) followed by incubation with DyLight 488 NHS ester (Pierce). Toggle-25 was titrated against 10^6^ cells in 200 μL TBB containing 0.1% BSA for 3.5 hours at room temperature. Cells were washed twice with TBB + 0.1% BSA prior to resuspension in 90 μL TBB followed by FACS analysis (BD Accuri C6 flow cytometer, FL1 channel).

To test binding of I-SELEX aptamer 5–12 to *t*RBCs, Toggle-25, *scr*Toggle-25 and 5–12 were synthesized with a 3′ 24 nucleotide (GAAUUAAAUGCCCGCCAUGACCAG)^44^ using PCR extension oligos CMB153, CMB154 and CMB152, respectively. A capture oligonucleotide (5′-biotin-CTGGTCATGGCGGGCATTTAATTC) complementary to the aptamer extension was synthesized and fluorescently labeled with streptavidin-phycoerythrin (SP). Aptamers were incubated with equimolar concentrations of capture oligonucleotide for 5 minutes at 75 °C then cooled slowly to 4 °C. Aptamer-capture oligonucleotide and SP were pre-complexed by incubating them in a 1:2 molar ratio for 15 minutes at room temperature. Excess biotin (20 μL biotin-saturated PBS, pH 7.4) was added for 15 min at room temperature to cap any free biotin-binding sites. The aptamer-capture oligonucleotide-SP complex (100 nM) or capture oligonucleotide-SP complex (100 nM) was incubated with either 10^6^ *t*RBCs or *s*RBCs in 100 μL TBB containing 1 μg BSA and 0.25 μg yeast tRNA for 1 hour at room temperature. Cells were washed with TBB and resuspended in 50 μL TBB prior to FACS analysis (FL2 channel).

### Bio-layer interferometry binding studies

Aptamer binding kinetics were determined using a BLItz machine and Streptavidin (SA) Dip and Read biosensors (Forte Bio). TBB was used for probe hydration (10 min), baseline readings, aptamer dilutions, and dissociation steps. Thrombin-BFPRck (Haematologic Technologies Inc.) at 10 nM was immobilized on a SA biosensor via a 300 s loading incubation. Aptamers were *in vitro* transcribed, DNase treated, and refolded as described above, then incubated with thrombin-loaded SA biosensors (association: 180 s; dissociation: 180 s) at various concentrations. Probes were regenerated using the following protocol: 60 s incubation in regeneration buffer (1 M NaCl, 1 mM NaOH) followed by 3 × 60 s incubations in TBB. The probe was regenerated at least once prior to collection of binding data using BLItz software for analysis.

### Parasite culture and preparation of *Plasmodium falciparum* infected RBCs (iRBCs)

*Plasmodium falciparum* CS2 and DC-J parasites were cultured at 10% hematocrit and 1–10% parasitemia in 5% O_2_ and 5% CO_2_ in RPMI-1640 media supplemented with 5 g/L Albumax II (Life Technologies), 25 mM HEPES pH 7.4 (pH adjusted with potassium hydroxide), 2 g/L sodium bicarbonate, 1 mM hypoxanthine and 50 mg/L gentamicin. DC-J parasites were cultured in media with a final concentration of 20 μg/mL blasticidin-S (Research Products International). Parasites were synchronized by incubation in 300 mM L-alanine, 10 mM HEPES for 5 minutes at 37 °C in order to lyse late-stage parasites. Uninfected human RBCs were mock cultured, “passaged,” and “synchronized” identically to iRBCs such that negative selections during I-SELEX also account for cell culturing effects.

### I-SELEX on malaria parasite-infected RBCs

Library preparation, amplification, and handling were identical to that of *tRBC* SELEX (described above). Malaria iRBC I-SELEX conditions were identical to those used in *t*RBC I-SELEX, with the exception of cell targets. Uninfected human RBCs (10^8^ per round) served as negative selection targets and were mock cultured under identical conditions to iRBCs. Late-stage (trophozoite and schizont), highly synchronized *Plasmodium falciparum* CS2 iRBCs were enriched to high (>90%) parasitemia using LD MACS® Columns (Miltenyi Biotec). 10^6^ iRBCs were used as positive selection targets. Five rounds of selection were carried out under conditions identical to *t*RBC I-SELEX. For Rounds 6–8, two selection streams were carried out in parallel. In selections 1 and 2, incubation of the library with target cells was carried out in 5 mL and 1 mL TBB, respectively. When partitioning Rounds 6–8 of selection 1 using the device, the sheath buffer volume was scaled accordingly from 10 mL to 50 mL. DNA from selection Rounds 8.1 and 8.2 were cloned into pGEM®-T vectors (Promega), transformed into DH5α cells and mini-prepped plasmid DNA sequenced to identify isolated aptamers (Supplementary Table S2).

### iRBC protease treatment

Late-stage CS2 iRBCs were first enriched by MACS® column separation as described above to >90% parasitemia. Cells (6 × 10^7^) were incubated with either Trypsin (Sigma) or Proteinase K (Sigma) at 1000 μg/mL in 1 mL of TBB for 30 minutes at 37 °C. Protease-treated cells were then washed 3x with 1 mL TBB, 10 μL protease inhibitor cocktail (Sigma).

Protease digestion of surface-exposed proteins was monitored by loss of anti-glycophorin A (GPA) antibody reactivity. PE-conjugated anti-GPA (25 ng per 10^5^ cells, Life Technologies) was incubated with untreated iRBCs, and trypsin- and proteinase K-treated in 200 μL TBB with gentle agitation for 30 minutes before being washed 3x with 200 μL TBB. Cells were resuspended in 200 μL TBB and analyzed on an Accuri C6 cytometer (BD Biosciences) in the FL2 channel. Fluorescence levels were compared to FL2 channel auto-fluorescence of untreated iRBCs without anti-GPA incubation.

### iRBC aptamer enrichment quantification and apparent K_*d*_ determination

Sequences from the terminal rounds of iRBC I-SELEX were tested for preferential binding to iRBCs over RBCs using the qPCR-based Enrichment Experiment protocol described above and is illustrated (Supplementary Figure S1). Briefly, 100 nM of a single aptamer species (for enrichment experiments) or a six-point titration of 0–100 nM aptamer (for apparent K_*d*_ determination) was incubated with either 10^7^ CS2 or DC-J iRBCs (MACS-enriched to >90% parasitemia) or uninfected, mock-cultured RBCs in 1 mL TBB for 60 minutes at room temperature before being passed through an I-SELEX device. 10 μL of the aptamer reaction (before addition of cells) was collected as to quantify aptamer “Input.” Cells were recovered directly from the sample outlet onto a vacuum filter plate membrane (Millipore MSHVS4510). Bound aptamers were eluted (off-vacuum) by a five-minute incubation with 1 mM EDTA in TBB, pH 7.4 followed by plate centrifugation. Aptamer “Output” eluted from the cell surface was concentrated by ethanol precipitation and resuspended in 8.5 μL TBB. The RNA Output was reverse transcribed using primer CMB183 and SuperScript III (Life Technologies) in accordance with the manufacturer’s instructions. Quantitative polymerase chain reaction (qPCR) with SYBR Green was used to determine relative levels of Input and Output aptamers as described above for different cell types using SELEX Forward and Reverse primers (Supplementary Table S3). For apparent K_*d*_ determination, aptamer binding to CS2 iRBCs is fit to the following model: [Bound Aptamer] = B_max_*[Aptamer] ⁄ ([Aptamer] + K_*d*_) + NS*[Aptamer] + background, and recovery against mock-cultured uninfected RBCs was fit using the nonspecific model [Bound Aptamer] = NS*[Aptamer] + background, where B_max_ is the concentration of binding sites, K_*d*_ is the apparent dissociation constant, and NS represents aptamer concentration-dependent nonspecific binding.

All aptamers and scrambled negative control sequences (Supplementary Table S2) were tested in triplicate in two independent experiments. The difference between Input and Output qPCR crossing points (ΔCp) was calculated for each iRBC sample and the compared to the ΔCp for mock-cultured RBC samples. The resulting difference between cell types (ΔΔCp) is used to quantify preferential aptamer enrichment for CS2 iRBCs over RBCs, protease-treated CS2 iRBCs, or DC-J iRBCs. Error was propagated when taking each difference and statistical significance (P < 0.05) was calculated using a Student t test.

## Additional Information

**How to cite this article**: Birch, C. M. *et al.* Identification of malaria parasite-infected red blood cell surface aptamers by inertial microfluidic SELEX (I-SELEX). *Sci. Rep.* **5**, 11347; doi: 10.1038/srep11347 (2015).

## Supplementary Material

Supplementary Information

Supplementary Information
