# Supplementary material for: Identification of malaria parasite-infected red blood cell surface aptamers by inertial microfluidic SELEX (I-SELEX)
Source: Sci Rep. 2015 Jul 1;5:11347. doi: 10.1038/srep11347 (PMC4486934; doi:10.1038/srep11347)
Supplement: Supplementary Information [file srep11347-s2.pdf]

Identification of malaria parasite-infected red blood cell surface aptamers by inertial microfluidic SELEX (I-SELEX)

Christina M. Birch[1], Han Wei Hou[2,3], Jongyoon Han[1,2,3] and Jacquin C. Niles[1]

[1]Department of Biological Engineering, Massachusetts Institute of Technology, 77 Massachusetts Avenue, Cambridge MA 02139, USA

[2]Department of Electrical Engineering and Computer Science, Massachusetts Institute of Technology, 77 Massachusetts Avenue, Cambridge MA 02139, USA

[3]BioSystems and Micromechanics (BioSyM) IRG, Singapore-MIT Alliance for Research and Technology (SMART) Centre, 1 Create Way, #04-13/14 Enterprise Wing, Singapore 138602, SINGAPORE

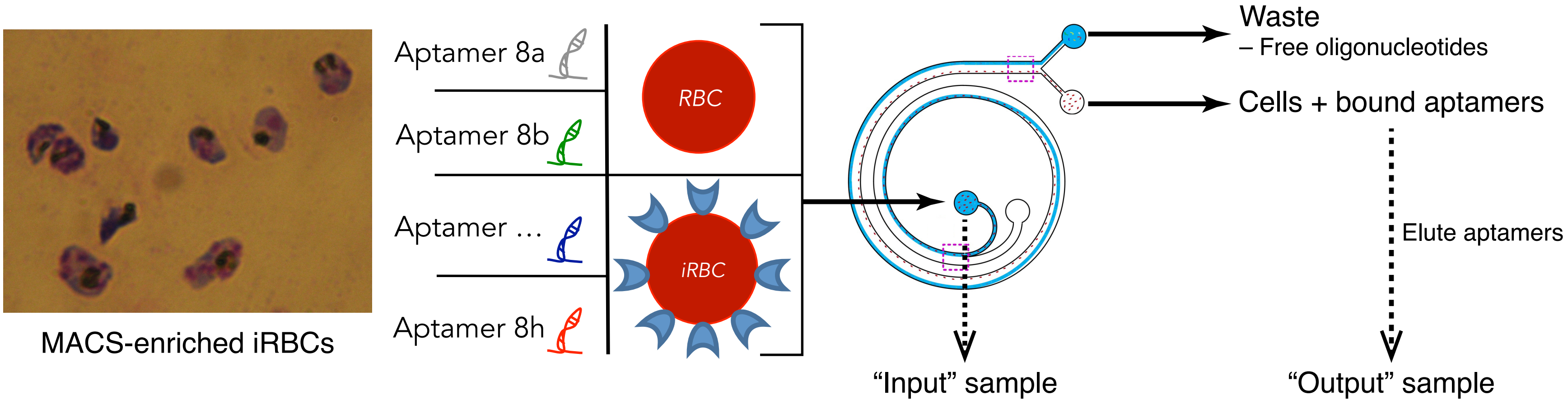

Supplementary Figure S1. A schematic of the aptamer enrichment assay. A single aptamer species is incubated with a target cell type (CS2 iRBCs, protease-treated iRBCs, DC-J iRBCs) or a negative control cell type (uninfected, mock-cultured RBCs). A small fraction of the reaction mixture is measured by qPCR to verify the “input” amount of aptamer. After incubation, the reaction mixture is passed once through the inertial microfluidic device, partitioning unbound aptamers from cells. Aptamers bound to the cell surface are eluted and quantified by qPCR as the “output” amount of aptamer. As measured by qPCR, all input samples have identical Crossing Points (Cp) and output samples are dependent on reaction conditions. The difference between input and output Cps (ΔCp) is used to calculate aptamer recovery. Aptamer recovery between target and control cell types (ΔΔCp) is used to establish statistical significance of aptamer enrichment.

Supplementary Table S1. Sequences recovered from de novo I-SELEX on tRBC targets.

Clone ID	K_d (nM)	Sequence of variable region
**Cluster I**		
3-25		**GUUACUG** AUCUUCCUGCAGCGCGAAUCACAUGUAUGAAGCCGGAUCGACG
3-30		A **GUUACUG** CGCUCUUACGAGGUAACUACUUAGUUGGCAUUACGUAGUACU
5-1		A **GUUACUG** AGCUCUUGUGUGUUACAGUUGAGAAUCACAACGAUUCCCUG
5-2		A **GUUACUG** GGUUCCCCUUCCCACACGCCAUCAUCGUAUGCCGGCAAACGA
**5-5**	2	A **GUUACUG** AGUUCCGGGGAGAGGGGUAGAGCUCUACCGCCCAAAUAGCGA
5-8		**GUUACUG** GGUUCCGCACAAAUGAGAUUUAUGUUUUUCUAAUCUGCCUCA
5-10		**GUUACUG** AGAGCCCUUGACCUCUGGAGCCCACGACGUCGUGAAUAUGAGG
5-11		**GUUACUG** ACACUCCCCGUUGGUGCGAAGCACCAAGUAACGACAGACUCAG
**5-12**	2	**GUUACUG** AGCUCAGUCGGGGUGACGCGUCACCCUCUAGGAGAGACUCUGU
5-14		A **GUUACUG** AUGUCCCCGAACUGGUGUGGCAAUGGCAGACAUUGCAGCAGG
5-15		A **GUUACUG** CGCUCCUAUACUGGCUCAUUGUGCCCGGCAUCGAGGACAUUG
5-17		**GUUACUG** AACUCUCGCCGUGGCGCCACGUUGAUAUGCAGUCGACUUCAAG
**Cluster II**		
**Toggle-25**	40	GA<u>ACAAAGCUGAAGUAC **UUA**</u>**CCCAAG** AUCAUCCCGA
3-4		UGAUGUAAGACUGUUACUUGUGUGUUA **UUGCCCAAG** UUUGGUCUGUAUUG
3-10		UGGUGACUGGGUGAGAAUAGCAUU **UUGCCCUAG** UCAUUCGAGUCUACAAC
**3-19** (2)	> 1000	CUGCCUGAGACUGCACUUUUUCGCUCC **UUCCCCAAG** UCUUUUUGCGAGUU
5-6		CGAAUGCCGAAAUUAGAGCGUG **UUCUCCAAG** CCCGACUCGUACGGCUCGA
**Cluster III***		
3-1		UCAAAUUAGUGCGCGAGGACUAUUAGGACGUCCAGUACAUAAUAUCAGAA
3-2		CGAAAAACAGUAUUACGAUGCAUCCUUCGUAAUCCACCUUGAAAUAAAAC
3-5		UAAGUUGCAGUAAUGGAGUUCCUGAAAUGGACGUACGGUCCUAUU
3-6		UAGUCAUUGGUUAAAAACCAGUCUCCAAUCGAAUAUUACCGGUUAUGUUU
**3-7** (4)	> 1000	CAACGGAUCACACGCAAGCGAAUCUAUGUCCUUCGGGUCUAAGGGUCGCU
3-8		UCCAUUUCUUCUGAAACGAUGAGCAC
**3-9**	4	ACGAACGUACAAGUUAUGAGAACUGACCUCCCUAUUGUCAUCCACACUCA
3-11		ACUUAAGAAUCGACCUGGGGUGACAAUUAAUCCUUCUUUGUUUUGUUAAC
**3-12**	200	GAUCAAAUUUAAUAUAUCCGAUCUGGAGAUCUAAUAUCAUAUUGUAGUGG
3-13		ACAGGUUUAUAAAGUUUUCGUUUCGAUGGAUUUUAAAACAAUCUAGAUCC
3-14		AUAAGCUGAUUUGUUCAGAACAUCUUAAUAUACAGUCACACAGGCUGCAG
3-15		CCAUUUUAAGGAAUCACGCUGCCCCUACCCUGCGGUUCAGCCGACCUUU
3-16		GGUUUUAAUUUAGUACUUCUCUAUAGUUUUACUAGCCUUGAACAUUGGGA
3-17		CUUUGCCUUACAUCAAUAUAGGUCAACAUAAGUAGUGUAACGUUUGGUCA
3-18		UACCUGUUAAGAAUGAGUUCAAGAGUAAAAGUUAUGUCCUGCAUGUCUAG
3-24		UGGGAGACCGAUAUAAGCAAUGUAUCACCUGUGCGCAUUGAAGUCGGACU
3-26		CAUUUGCGCGAAUCAGGCUUGCUUGUGUUAACACUGCAACGGUUUUAUCG
3-27		CCGCGGACUUGCACUGUACCGCGUACCUUGCCUAAUUGUUCUGUAAAGAA
3-28		AAGGUUGAUGGCGUUGAUUCUUAGUCAUUAAUCGUUGUCGUUAAAGACGU
3-29		AUGAUUGAUUAUCAUGAAAAUGGCUGGUGGUAAACAUCACCUGAUAUUG
5-7		UUGCACCAAGGUUCGGAAAUUGAGCGGGCGACGCGUCGAAUGCCGCUAAU

Supplementary Table S2. Sequences recovered from de novo I-SELEX on malaria-parasite infected red blood cell targets. Clones are grouped by the stringency of the selection from which they were derived: Selection 1 (increased stringency for Rounds 6-8) and Selection 2 (consistent stringency across all 8 rounds). Tested sequences are shown in bold alongside their enrichment values (ΔΔCp) and the statistical significance (P value) of enrichment over scrambled negative control sequences (scr8.1-1 and scr8.2-1).

Clone ID	Copy #	ΔΔCp	P value	Sequence of variable region
**Selection 1**				
**8.1-1**	4	3.115	0.0002	CAACGGATCACACGCAAGCGAATCTATGTCCTTCGGGTCTAAGGGTCGCT
**8.1-2**	2	3.405	0.027	GTCAGAGTATGGGTACTGCAAGCTGCATGTGTTGCTCCGCTTAGACCCTA
**8.1-3**	1	1.476	> 0.1	TCCCGCGGCCATGGCGGCCGGGAGCATGCGACGTCGGGCCCAATTCGCCC
**8.1-4**	1	1.965	> 0.1	GTCCGGGTAGCGAGAGGACTTGCGACGTTTGGTGAGCATAAGCTGTAGAC
**8.1-5**	1	3.18	0.0378	GTAAGCGGACGATGGTGCGTGGGCGAATTGATCAGTACTTACTGTGCGAA
8.1-6	1	-	-	GTGGAATACAACCCCGACTTAAAAACCTAACCGGATTCCGTTTGGCGCAA
8.1-7	1	-	-	GGCATCGATTGTATTACTCATGTCCTCCGAGTACTCCGAATGGCCGGCGT
8.1-8	1	-	-	GTGAGTGTCGTTTGACAATCGGAGCTAAGATGATGGCCGTGGTCAACGGA
8.1-9	1	-	-	TGTTAGAGATTTCTTGGAGCGTCGAAAGTAGGATGCCAAGATCGGTTGGA
8.1-10	1	-	-	GAGTGGTATCGGATCGGACGAATAGATTGCCGCGTTAGGATCGTACCTGA
8.1-11	1	-	-	TTTGGAGCGATATGCGATATATCGATTGTAGATGAAGTCATGCAGTGGAA
8.1-12	1	-	-	GACAAAGATCCTAAAACATGCGCGTAACCGGACAGTATTGTAGCTTTGTG
**Selection 2**				
**8.2-1**	3	2.631	0.0159	CCAGGGGTAAGTTAGTGTAGGACTTGTTCTACATAGGATCGAATCGGTGG
**8.2-2**	3	3.388	0.0243	CTCGGATACTTAATAATAGAATTGGCCCAGACGCATGCATGATCCGCCAG
**8.2-3**	1	3.125	0.0131	GGCCTATCTGGGAGGAAAGCAGCCCAATTCGAACAGGTATGTGTGATCTG
8.2-4	1	-	-	AGGAAACTAATGTTGATACAACTGGGTACAATTAGTGATCCCGGTCATCT
8.2-5	1	-	-	CTTTTGTTGTGATCGTTTATCCTTAATGGTTTATCCGCAGCTGGGGATA
8.2-6	1	-	-	GGAGTATCAATATCGGCACAGGTATGAGCAATCAGTTATGCACGTTTCGC
8.2-7	1	-	-	GGGGATTGGGTGTGCAGTCCTGTAGAGTAGGTTCACGTAGTTAGCGTTCA
8.2-8	1	-	-	AATTGGTCCGAATGTACGGAGTTTTCGTCTAGATGCTAGGAAATGGTGCC
8.2-9	1	-	-	GTATAGGGTACTGTTCCTGCTGCATTGGGCAGGTCATATAAGTCAGTTCT
8.2-10	1	-	-	TGGTGGTCGTCCTCAAAAACCGCCTATTTGCGATGGATAGGCGTATTGCTC
8.2-11	1	-	-	GCAATGATTTGCATATCCTTCTGAGCTGTGATCACGCTAACATAACAGTC
8.2-12	1	-	-	TGACTTAAATGCCGAAGCGTTGACGTGATGGAGGATGTTGCGAACATTTC
8.2-13	1	-	-	TGAAAGCACTACATTTATTGGTGATGAAAGTGCGTGATGCTGTGTTCCGC
**Scrambled Sequences**				
**scr-8.1-1**	n/a	1.243	null	ATGTCTCCAGCTCTTGTGGCGAACCCAAGCGGGTTGACCAGAATGCATCA
**scr-8.2-1**	n/a	1.302	null	GAATGGTGTATATGTTGACAGTCAAGCGTCTCAGGTGCGGTAAGTCAGGT

Supplementary Table S3. **List of primers used in this work.**

CMB49	CCGCTCGAGTAATACGACTCACTATAGGGAGAGAGGAA
CMB50	CTTCAGCTTTGTTCCCCATCCCTCTTCCTCTCTCCCTATAGTGAGTC
CMB51	GATGGGGAACAAAGCTGAAGTACTTACCCAAGATCATCCCGAACGA
CMB56	TCGACCTCTGGGTTATGTCGTTCGGGATGATCTTGGG
CMB77	GGGAGAGAGGAAGAGGGATGGG
CMB94	TCGACCTCTGGGTTATGTCGTTC
CMB95	CCGCTCGAGTAATACGACTCACTATAGGGAGACAAGAT
CMB96	GCAGCGTTCCTCGATGGCCTTGATCTTGTCTCCCTATAGTGAGTCG
CMB97	CATCGAGGAACGCTGCCTACACACATGGACATAAACAAGAGCCGA)
CMB98	CCATTTTCTCGCCCTCTTCGGCTCTTGTTTATGTCCATG
CMB104	TTCTCGCCCTCTTCGGCTCTTGTTTA
CMB106	GAGACAAGATCAAGGCCATCGAGGAA
CMB107	CACTATAGGGAGCTCAGAATAAACGCTCAAACGAACGTACAAGTTATGAGAACTG
CMB108	CGAATGAGTGTGGATGACAATAGGGAGGTCAGTTCTCATAACTTGTACGTTCGT
CMB109	GCCGGATCCGGGCCTCATGTCGAATGAGTGTGGATGACAATAGG
CMB112	CACTATAGGGAGCTCAGAATAAACGCTCAACAACGGATCACACGCAAGCGAA
CMB113	GCGACCCTTAGACCCGAAGGACATAGATTCGCTTGCGTGTGATCCGTTGTTG
CMB114	GCCGGATCCGGGCCTCATGTCGAAAGCGACCCTTAGACCCGAAGGACATAGA
CMB116	AGTCTCTCCTAGAGGGTGACGCGTCACCCCGACTGAGCTCAGTAACTTGA
CMB117	GCCGGATCCGGGCCTCATGTCGAAACAGAGTCTCTCCTAGAGGGTGACGCGT
CMB119	CGAACCACTACAATATGATATTAGATCTCCAGATCGGATATATTAAATTTGATCTTG
CMB120	GCCGGATCCGGGCCTCATGTCGAACCACTACAATATGATATTAGATCT
CMB122	CTCGCAAAAAGACTTGGGGAAGGAGCGAAAAAGTGCAGTCTCAGGC
CMB123	GCCGGATCCGGGCCTCATGTCGAAAACTCGCAAAAAGACTTGGGGAAGGA
CMB125	TCGCTATTTGGGCGGTAGAGCTCTACCCCTCTCCCCGGAACTCAGTAACTTTGAG
CMB126	CACTATAGGGAGCTCAGAATAAACGCTCAAGTTACTGAGCTCAGTCGGGGT
CMB127	GCCGGATCCGGGCCTCATGCTGAGCTCAGTAACTTGAGCGTTTATTCTGAGCTCCCTAT
CMB130	CACTATAGGGAGCTCAGAATAAACGCTCAAACCGATGAGCTCAGTCGGGGT
CMB131	AGTCTCTCCTAGAGGGTGACGCGTCACCCCGACTGAGCTCATCGGTTTGA
CMB132	GCCGGATCCGGGCCTCATGTCGAAACAGAGTCTCTCCTAGAGGGTGACGCGT

CMB133	ATAGGGAGCTCAGAATAAACGCTCAAGATCAAATTTAATATATCCGATCTGGAGATCTAA
CMB134	CTATAGGGAGCTCAGAATAAACGCTCAACTGCCTGAGACTGCACTTTTTCG
CMB139	CTCTCCCCGGAACTCATCGGTTTTGAGCGTTTATTCTGAGCTCCCTATAGTG
CMB140	TCGCTATTTGGGCGGTAGAGCTCTACCCCTCTCCCCGGAACTCATCGGTTTTGAG
CMB141	GCCGGATCCGGGCCTCATGTCGAATCGCTATTTGGGCGGTAGAGCTC
CMB142	CACTATAGGGAGCTCAGAATAAACGCTCAAAGTTACTGAGTTCCGGGGAGAG
CMB152	CTGGTCATGGCGGGCATTTAATTCGGGCCTCATGTCGAAACAGAG
CMB153	CTGGTCATGGCGGGCATTTAATTCTCGACCTCTGGGTTATGTCGTT
CMB154	CTGGTCATGGCGGGCATTTAATTCCCATTTTCTCGCCCTCTTCG
CMB183	GCGGCTGTCTCCACAAGTCGCCGGATCCGGGCCT
SELEX Library template	CCGAAGCTTAATACGACTCACTATAGGGAGCTCAGAATAAACGCTCAA-[N_{50}]-TTCGACATGAGGCCCGGATCCGGC
SELEX Forward Primer	CCGAAGCTTAATACGACTCACTATAGGGAGCTCAGAATAAACGCTCAA
SELEX Reverse Primer	GCCGGATCCGGGCCTCATGTCGAA
Capture Oligo	Biotin-CTGGTCATGGCGGGCATTTAATTC
